# Reliability and validity of the short version of the childhood abuse self report scale in Chinese college students

**DOI:** 10.1186/s40359-024-01734-5

**Published:** 2024-05-08

**Authors:** Yali Zhang, Jinxia Zhao, Yuewen Bian, Fuhai Zhang

**Affiliations:** College of Education, Hebei Normal University, Shijiazhuang, China

**Keywords:** Childhood abuse, Childhood maltreatment, Reliability, Validity, College students

## Abstract

**Background:**

The reliability and validity of the current scale for measuring childhood abuse in China are worrying. The development of the Short Version of the Childhood Abuse Self Report Scale (CASRS-12) helps to change this situation, but the effectiveness of the tool has not yet been tested in Chinese participants. This study aims to test the reliability and validity of the CASRS‑12 in Chinese college students.

**Methods:**

A total of 932 college students were investigated, of whom 418 were investigated for the first time, and only the CASRS‑12 was filled out. In the second survey, 514 participants filled out the CASRS‑12, Depression Scale, Self-esteem Scale and Subjective Well-being Scale in turn. After 4 weeks, 109 participants were selected for retest.

**Results:**

Each item of the CASRS‑12 had good discrimination. Exploratory factor analysis and confirmatory factor analysis (χ^2^/df = 4. 18, RMSEA = 0. 079, CFI = 0. 95, TLI = 0. 94, IFI = 0. 95, NFI = 0. 94) all supported the four-factor structure of the scale, and the cumulative contribution rate of variance was 76.05%. Cronbach’s α coefficient and retest reliability were 0.86 and 0.65, respectively. Childhood abuse was positively correlated with depression (*r* = 0. 42, *p* < 0.01), and negatively correlated with self-esteem (*r*=-0. 33, *p* < 0.01) and subjective well-being (*r*=-0. 32, *p* < 0.01).

**Conclusion:**

The Chinese version of CASRS‑12 meets the measurement standard and could be used to measure the level of childhood abuse of Chinese college students.

## Introduction

Childhood abuse refers to the behavior committed by a person who has a duty of care and supervision of a minor (before the age of 18), which are likely to cause actual or potential harm to his or her health, development and dignity, including psychological abuse, sexual abuse, physical abuse and neglect [1]. Meta-analysis found that the detection rates of physical abuse, emotional abuse and sexual abuse among primary and secondary school students in China were between 12% and 30%, but the detection rates of physical neglect and psychological neglect reached 47% and 44% respectively [2]. Looking at other countries, the incidence of abuse, especially neglect, has remained high in recent years. For example, recent studies have found that the incidence of abuse within one year is as high as 89.9% in India [3] and 69.6% in Ecuador [4]. It can be seen that childhood abuse has become a global problem that needs to be solved urgently. Additionally, Childhood abuse will not only cause internalized problems such as anxiety, depression and sleep disturbance [5–7], but also lead to externalized problems such as aggressive behavior, opposing defiance, addiction behavior, and even self-harm and suicide [8–10], and this effect may exist for a long time.

In view of the prevalence of childhood abuse and its extensive and long-term impact, many researchers and policy makers are committed to exploring the key influencing factors and monitoring the incidence of childhood abuse dynamically in order to better prevent and monitor it. In terms of influencing factors, the current research mainly discusses the inducing and protecting effects of societal, community, interpersonal, and individual factors on childhood abuse based on the framework of ecosystem theory. By analyzing societal factors, it was found that economic recession and social norms about gender inequality can induce childhood abuse, while paid parental leave, increases in minimum wage, and more generous welfare benefits may prevent childhood abuse [11, 12]. The analysis of community factors found that neighborhood crime and socioeconomic disadvantage increase the stress level of parents and families, erode the social network, and in turn, contribute to the risk of child maltreatment. In contrast, providing more health, social, and educational services in the community is an important reason to prevent childhood maltreatment [13]. The analysis of interpersonal factors showed that family poverty, parents’ mental health and drug use disorders, and intimate partner violence are risk factors for childhood abuse, while social support can reduce the stress of caregivers, improve their well-being, and reduce the risk of abuse [14]. By analyzing individual factors, it is found that young age and special medical needs or disabilities are risk factors for abuse, while self-regulation skills, social skills, adaptive function, and self-esteem are protective factors of abuse [15].

The discussion about childhood abuse goes beyond this, and its measurement has become a key issue. Currently, abuse screening is mainly conducted using questionnaires, and the most commonly used one is the Childhood Trauma Questionnaire (CTQ). It consists of 25 questions, scored on a 5-point scale, and includes 5 dimensions of emotional abuse, physical abuse, sexual abuse, physical neglect, and emotional neglect [16]. In addition, the Child Abuse Self-Report Scale (CASRS) is commonly used in previous studies. It has 38 questions, using a 4-point score, including 4 dimensions of emotional abuse, physical abuse, sexual abuse, and neglect [17]. However, it is found that there are many shortcomings in the above tools. First of all, the number of questions is more than 20, and the similarity among multiple questions is too high, which seriously increases the burden and boredom of the participants and reduces the test efficiency. Secondly, the reliability and validity are worrying. Except CASRS, the reliability of CTQ in a few dimensions is not ideal, especially the structural validity is poor. This is because the scale contains items that are not suitable for the current era or cross-cultural background, such as “Parents don’t let me eat enough”. In addition, under the background of the gradual advancement of longitudinal study, an efficient, concise and culturally adaptable questionnaire is necessary.

In view of this, Fekih-Romdhane et al. Took the full version of CASRS as a blueprint, deleted some outdated items based on Asian cultural background, and merged some highly similar items to form CASRS-12 [1]. The revised questionnaire has good reliability and validity, and still retains the original four-factor structure, but the number of questions has changed to 12, which greatly improves the test efficiency. Up to now, there is still a lack of concise and efficient tools to measure childhood abuse in China. Therefore, research tools have always been one of the puzzles that limit the further development of research in this field in China. Consequently, this study intends to translate and revise the CASRS‑12 based on Chinese college students. Since childhood abuse is closely associated with depression, self-esteem, and well-being, this study identified it as a criterion variable to verify the validity of CASRS‑12.

## Methods

### Participants

Sample 1 (For item analysis and exploratory factor analysis): Using convenience sampling, six classes were selected, and students were invited to fill in online surveys and forward online links voluntarily. Each participant who carefully answers the questions can get a 5-yuan fee. After eliminating invalid data (such as incomplete filling and filling in the same option), 418 valid participants remained (186 boys, 232 girls; 121 freshmen, 114 sophomores, 87 juniors, 96 seniors; 189 only children, 229 non-only children; Mean age 19.24 ± 2.13 years).

Sample 2 (For confirmatory factor analysis and criterion validity analysis): 12 classes majoring in literature and history, science and engineering and art were selected in three universities in Hebei Province by convenient sampling, and 578 questionnaires were distributed. After eliminating invalid questionnaires (such as incomplete filling and filling in the same option), 514 questionnaires were retained (223 boys and 291 girls; 197 freshmen, 165 sophomores, 152 juniors; 321 students in towns and 193 students in rural; Mean age 19.93 ± 1.67 years).

Sample 3 (For test-retest reliability): Three classes were randomly selected from sample 2, and the initial test questionnaire was distributed online again after one month. The number of valid participants in both tests was 109 (42 boys, 67 girls; 36 freshmen, 45 sophomores, and 28 juniors).

### Instruments

#### Shortened version of the childhood abuse self report scale (CASRS‑12)

This scale was developed by Fekih‑Romdhane et al. [1] and contained 12 items to measure physical abuse, psychological abuse, sexual abuse, and neglect. Items are scored on grades 0 ∼ 3 (never ∼ always). The higher the total score, the more serious the childhood abuse. After the developer of the original scale allows the revision. First, three masters of psychology independently translated English into Chinese. Subsequently, they discussed the controversial and ambiguous places and formed an initial questionnaire. Thirdly, an English major student and a psychology teacher with a background in studying abroad back-translated it into English, compared the differences between the back-translated version and the original version, discussed and revised the differences, and finally formed the Chinese version.

#### Depression scale

This scale was derived from the depression subscale in the short version of Self-Rating Symptom Scale [18], with 7 items (e.g., “I feel depressed and sad”), which is a one-dimensional structure. It is scored from 0 to 4 (not at all to severe), with higher scores indicating more severe depression. The Cronbach’s α coefficient of this scale was 0.84.

#### Self-esteem scale

It was revised by Chang et al. [18], which measures the general self-esteem level. It is a one-dimensional structure and consists of five items (e.g., “I have a positive attitude towards myself”). All items are scored from 1 to 4 (strongly disagree to strongly agree), with higher scores indicating a higher sense of self-worth. The Cronbach’s α coefficient of this scale was 0.89.

#### Subjective well-being scale

It measures the general level of well-being and consists of three items (e.g., “I am very happy”), which is a one-dimensional structure [19]. Items are scored from 1 to 4 (never to always), with higher scores indicating greater happiness. The Cronbach’s α coefficient of this scale was 0.85.

### Data analysis

SPSS26.0 was used for descriptive statistics, correlation analysis, and exploratory factor analysis, and AMOS24.0 was used for confirmatory factor analysis.

## Results

### Item analysis

The correlation between each item and the total score of the scale was investigated by item analysis. The results showed that the discrimination of each item ranges from 0.44 to 0.66, all of which are above 0.4, indicating that the discrimination of the items is good, and all the items are retained (Table 1).

### Exploratory factor analysis

The results showed that the KMO value was 0.81 and the Bartlett’s sphericity test value was 2496.61 (*p* < 0.001), which indicated that it was suitable for factor analysis. Then, the principal component analysis and Promax rotation were used to extract factors with eigenvalues greater than 1. In addition, the screening of factors and items should meet the following criteria: (a) the commonality of each item should be higher than 0.4. (b) The factor loading of the item is unique, and the difference in cross-factor loading values of different items needs to be higher than 0.05; (c) Factor naming is easy. Based on the above criteria and combined with the scree plot, 4 factors were finally extracted, and all items were retained. The loadings of each item within the factor exceeded 0.8 (*p* < 0.01), and the cumulative explanation rate was 76.05%. The four factors were named psychological abuse, sexual abuse, neglect abuse, and physical abuse (Table 1).

**Table 1:** The discrimination and factor loading for each item of CASRS‑12

Items	Discrimination	Factor 1	Factor 2	Factor 3	Factor 4
1. My parents treat me with disrespect.	0.66	0.88			
2. I feel worthless because of the way my parents treat me.	0.63	0.82			
3. My parents blame me in others’ presence.	0.53	0.86			
4. An adult of some people talk to me nastily.	0.44		0.90		
5. An adult or some adults have tried to touch my private part.	0.45		0.82		
6. An adult made me look at or touch his/her private parts.	0.46		0.90		
7. My family doesn’t pay attention to my wishes.	0.57			0.80	
8. I am allowed to decide for my wishes.	0.54			0.94	
9. I don’t spend a restful life.	0.47			0.90	
10. When my parents punish me, it is not proportionate to my mistakes.	0.55				0.87
11. I testify other members of my family are being beaten up.	0.47				0.83
12. If I do not obey the rules of my family, I will be punished very hard.	0.53				0.85
Eigenvalue		1.01	2.01	4.68	1.42
Variance (%)		8.44	16.78	38.97	11.86

Note: Factor 1 psychological abuse, Factor 2 is sexual abuse, Factor 3 is neglect, Factor 4 is physical abuse

### Construct validity analysis

Confirmatory factor analysis was carried out on the data of sample 2. The model was constructed based on the four factors obtained by exploratory factor analysis. The results showed that the model fitted well and the fitting indexes all met the measurement standards (χ^2^/df = 4.18, RMSEA = 0.079, CFI = 0.95, TLI = 0.94, IFI = 0.95, NFI = 0.94). It showed that CASRS‑12 is a four-factor structure.

### Criterion validity analysis

The total score of CASRS-12 was positively correlated with depression (*r* = 0. 42, *p* < 0.01), and negatively correlated with self-esteem (*r*=-0. 33, *p* < 0.01) and subjective well-being (*r*=-0. 32, *p* < 0.01) (Table 2).

**Table 2:** Correlation between CASRS‑12 and criterion variable (*n* = 514)

variables	1	2	3
1. Childhood Abuse	-		
2. Depression	0.42^***^		
3. Self-esteem	-0.33^***^	-0.42^***^	
4. Well-being	-0.32^***^	-0.44^***^	-0.43^***^

Note: ^***^p < 0.001

### Convergent validity and discriminant validity analysis

Convergence validity is used to evaluate whether the questions measuring potential traits belong to the same factor, which is often evaluated by average variance extraction (greater than 0.5 is good, 0.36 ∼ 0.5 is acceptable), factor loading (greater than 0.7 is good, 0.6 ∼ 0.7 is acceptable), and construct reliability (greater than 0.7 is good, 0.6 ∼ 0.7 is acceptable) [20]. The results showed that the average variance extraction from each factor was above 0.5, the factor loading of each item was above 0.6, and the construct reliability of each factor was above 0.7 (Table 3). Taken together, the convergent validity of CASRS‑12 is good.

**Table 3:** Convergent validity of CASRS‑12

factors	factor loading	average variance extraction(AVE)	construct reliability
1. Psychological abuse	0.72 ∼ 0.87	0.61	0.82
2. Sexual abuse	0.74 ∼ 0.86	0.68	0.86
3. Neglect	0.68 ∼ 0.96	0.69	0.87
4. Physical abuse	0.67 ∼ 0.87	0.62	0.83

Discriminant validity means that there are significant differences among the dimensions of a potential trait. If the AVE of the potential factor is greater than the square of the correlation coefficient between the factors, it indicates that the discriminant validity is good [20]. The results showed that the square of the correlation coefficients between each factor was smaller than the corresponding average variance extraction, indicating that the discriminant validity among the factors of CASRS-12 was good (Table 4).

**Table 4:** Discriminant validity of CASRS‑12

factors	correlation(r)	r^2^	AVE compared with r^2^
Psychological abuse - Sexual abuse	0.41	0.17	AVE_1_> r^2^, AVE_2_> r^2^
Psychological abuse - Neglect	0.47	0.22	AVE_1_> r^2^, AVE_3_> r^2^
Psychological abuse - Physical abuse	0.61	0.37	AVE_1_> r^2^, AVE_4_> r^2^
Sexual abuse - Neglect	0.16	0.03	AVE_2_> r^2^, AVE_3_> r^2^
Sexual abuse - Physical abuse	0.43	0.18	AVE_2_> r^2^, AVE_4_> r^2^
Neglect - Physical abuse	0.28	0.08	AVE_3_> r^2^, AVE_4_> r^2^

### Reliability analysis

Based on sample 2, the Cronbach’s alpha coefficient of CASRS-12 was 0.86, and the Cronbach’s alpha coefficient of psychological abuse, sexual abuse, neglect, and physical abuse were 0.82, 0.85, 0.86, and 0.84, respectively. The McDonald’s omega coefficient of CASRS-12 was 0.86, and the McDonald’s omega coefficient of psychological abuse, sexual abuse, neglect, and physical abuse were 0.82, 0.86, 0.85, and 0.83, respectively. Based on sample 3, the test-retest reliability of CASRS‑12 was 0.65, and the test-retest reliability of each dimension ranged from 0.57 to 0.68.

## Discussion

This study is the first time to test the reliability and validity of the shortened version of CASRS-12 in Chinese college students, which provides a practical tool for further exploration in this field in China. The results showed that the Chinese version of CASRS-12 had good discrimination, and the item-total correlation of each item was above 0.4, meeting the measurement standards [20], so all items were retained. Follow-up exploratory factor analysis showed that four factors (psychological abuse, neglect, physical abuse and sexual abuse) could be extracted from all items, which corresponded to the dimensions of the items in the original scale. The factor loading of each item in this scale is above 0.8, and the explanation rate is over 50%, which indicates that the scale can well measure the degree of abuse experienced in childhood.

At the same time, in terms of the four factors, the explanation rate of neglect is the highest, which shows that childhood abuse in current family education is mainly manifested in neglect. At present, when the material conditions are sufficient, neglect often has new manifestations, such as parental phubbing [21]. Confirmatory factor analysis also supported the four-dimensional structure of the scale, proving the cross-cultural stability of the structure of CASRS‑12. Additionally, it is worth noting that physical neglect and psychological neglect were measured separately in previous studies, and the combined measurement in this study can reduce the number of items, which also indirectly shows the irrationality of separating psychological neglect from physical neglect in the past, and the difference between them is low. The results are helpful to promote the enrichment of individual-centered research in this field to distinguish the sub-groups of childhood abuse in the new period, and provide tool support for in-depth understanding of the basic situation and characteristics of childhood abuse.

The results of criterion validity analysis found that CASRS‑12 is closely related to depression, and the strength of the correlation is moderate, which is consistent with previous studies [1], indicating that the scale is still effective after the number of items is reduced. At the same time, it also showed that individuals who have been abused in childhood may affect the satisfaction of their basic psychological needs, which may easily lead to an increased risk of depression [1]. In addition, this study found a significant negative correlation between CASRS‑12 and self-esteem, which is consistent with previous results [22]. This is because the degree of social support felt by abused individuals will decline, which will affect the acquisition of individual self-worth and greatly reduce the level of self-esteem [22]. This study also showed that there is a significant negative correlation between CASRS-12 and subjective well-being, and the more experiences of childhood abuse, the lower the level of well-being, which is consistent with previous research results [23]. The abuse experience encountered by children and adolescents during their growth will lead to psychological trauma, especially the diffusion of fear and the decline of interpersonal trust will reduce the level of individual happiness [24]. In a word, as in previous studies, CASRS-12 is correlated with self-esteem, depression and well-being to a similar extent, which indicates the effectiveness of this scale to some extent.

The results of convergent validity analysis found that the items under each factor can be effectively aggregated into the same structure, which indicates that the measurement content of each factor has a high degree of internal consistency. In addition, the results of discriminant validity analysis found that there were significant differences among different factors, which indicated that there were typical differences and unique characteristics among the four factors of child maltreatment. Additionally, reliability analysis showed that the reliability of psychological abuse, neglect, sexual abuse and physical abuse in Chinese version of CASRS-12 were all above 0.7, even exceeding 0.8, which also met the measurement standards [20]. It indicated that the items under each factor can be closely clustered together to reflect different aspects of childhood abuse. Finally, the test-retest reliability of the Chinese version of CASRS-12 within one month is above 0.6, indicating that the scale is relatively stable across time and is less affected by accidental factors. In short, the introduction of CASRS-12 is a helpful supplement to the measurement tools in the field of childhood maltreatment in China. It not only has fewer items but also has good reliability and validity, which is especially suitable for the current longitudinal studies.

## Conclusion

The Chinese version of CASRS‑12 meets the measurement standards and could be used to measure the level of childhood abuse of Chinese college students.

### Implications and limitations

This study has theoretical value. For the first time, we test the reliability and validity of CASRS-12 from many aspects in Chinese cultural context, prove its cross-cultural applicability, and increase confidence for the popularization of this scale in the world. Secondly, this study provides enlightenment for re-understanding the dimension of childhood abuse at present. As far as neglect is concerned, there may be no obvious distinction between physical neglect and psychological neglect at present. Finally, this study shows that the reliability and validity of CASRS-12 surpasses CTQ and CASRS, which indicates that it is necessary to pay attention to the improvement of the original tool and its performance. In addition, this study has practical value. The Chinese version of CASRS-12 provides a more efficient and rigorous measurement of childhood abuse in China, and also provides a reference tool for large-scale tests and longitudinal studies to measure childhood abuse.

The study also has some limitations. This study does not give the grading standard of childhood abuse, and the severity level of abuse can be given in combination with the frequency of abuse in the future. In addition, this study has not sampled all over China, so we can consider collecting more participants in different regions of China in the future to explore the norm level of childhood abuse in the new era.

## Data Availability

The datasets are available from the corresponding author on reasonable request.
